# Retraction: Comparing the predictive value of quantitative magnetic resonance imaging parametric response mapping and conventional perfusion magnetic resonance imaging for clinical outcomes in patients with chronic ischemic stroke

**DOI:** 10.3389/fnins.2024.1504798

**Published:** 2024-10-07

**Authors:** Frontiers Editorial Office

The journal retracts the article cited above.

Following publication, concerns were raised regarding the data used in the study. Specifically, during the investigation, which was conducted in accordance with Frontiers' policies, the authors failed to provide appropriate authorization for use of the clinical data that supports their study and failed to satisfactorily answer concerns over the misrepresentation of this data. Unauthorized data use and the misrepresentation of data are breaches of Frontiers' guidelines and those of the Committee on Publication Ethics. As such, this article is being retracted.

This retraction was approved by the Chief Executive Editor of Frontiers. The authors do not agree to this retraction.

